# The Impact of Multidimensional Health Levels on Rural Poverty: Evidence from Rural China

**DOI:** 10.3390/ijerph19074065

**Published:** 2022-03-29

**Authors:** Xueyan Chen, Tao Zhou, Di Wang

**Affiliations:** 1 School of Management Science and Real Estate, Chongqing University, Chongqing 400044, China; xueyanchen@cqu.edu.cn (X.C.); diwang@cqu.edu.cn (D.W.); 2 Research Center for Construction Economy and Management, Chongqing University, Chongqing 400044, China

**Keywords:** individual health, health-care system, rural poverty, spatial analysis

## Abstract

Poor health and poverty interact and restrict each other. While this relationship is acknowledged, little is known about the extent of its impact. By integrating multisource data, this study used spatial econometric models to quantitatively reveal the relationship between health and rural poverty and explore its intrinsic mechanisms. The results indicated that health-care system input, individual health status, and individual health-seeking behavior have a significantly positive effect on the eradication of rural poverty. The health-care system input is characterized by spatial spillover, significantly contributing to rural poverty alleviation in the region and neighboring regions, as well. However, the effect of health-care system services’ capability was negative. Thus, it is necessary to increase investment in the health-care system and pay attention to both the health status and healthy behaviors of rural residents. Moreover, further effort should be given to the supply-side reform of health services as a breakthrough point.

## 1. Introduction

Eradicating poverty and narrowing disparities has always been a global problem. Global poverty reduction faces challenges, such as the increase in poor people, and the outbreak of COVID-19, which have slowed down or even worsened the rate of poverty reduction. Rural poverty is even worse, with 80% of the world’s poor living in rural areas [1]. Facing the United Nations’ goal of sustainable development by 2030, it is imperative to return to the formation mechanism of poverty and to the question of how to alleviate poverty. Poverty caused by disease is considered one of the root causes of rural poverty in China, where 42% of the total of 70 million poor on record were trapped in poverty due to disease [2,3]. A better understanding of the relationship between rural residents’ health levels and poverty is important for developing more precise poverty alleviation policies.

A strong link between poor health and poverty is well established. Earlier studies focused only on the relationship between poverty and specific diseases (AIDS, tuberculosis, malaria, etc.) [4,5] and examined the relationship between health-care expenditures and poverty [6]. Clear connections between economic resources and health expenditures have been shown in both low-income and high-income countries [7]. When an illness occurs, the family’s medical expenses increase, their income decreases, savings are used and even worse, loans are taken from friends and relatives and the family property is sold, which means the loss of household physical capital [8]. A community survey in China shows that more than 36% of respondents have forgone health care in the past year due to high costs [9]. In the past two decades in China, the incidence of catastrophic health expenditures (CHEs) in rural areas has been higher than that of urban residents [10]. Additionally, poor health can also damage the accumulation of human capital in the longer term. It can limit labor productivity and add a budget constraint on skills training, which reduces the likelihood of increased income, securing the vicious cycle of disease and poverty [11]. For children, poor health may affect physical and mental development [12,13], further affecting educational attainment and future earnings [14,15,16]. Such health deficits may even have intergenerational effects, and the vicious circle of health–poverty in families is intensified [17]. Moreover, the contribution of improvements in health to the reduction of rural multidimensional poverty was more than 50% [18].

The World Health Organization defined health in terms of three dimensions of the individual: physical health, mental health, and the state of complete social adaptation [19]. Thus, residents’ health awareness and specific behavioral preferences should not be ignored. Poverty can cause stress and negative emotional states which in turn may lead to short-sighted and risk-averse decision making, possibly by limiting attention and favoring habitual behaviors at the expense of goal-oriented ones, ultimately making it difficult to eradicate poverty [20]. Poor people tend to lack foresight in nutrition and health management and are prone to make short-sighted decisions, meaning that they have a lower willingness to make a risky payment, which makes disease more likely to occur or for the best time to treat it to be missed [21]. Research proves that negative emotions, low health literacy, and unscientific health-care practices are indirect causes that reinforce the cycle between health and poverty [22].

On the other hand, poverty is a social concept, and analyzing its relationship with poverty in terms of specific or constrained health indicators alone is limited. While health refers to individual health, it also involves health service. The latter also has a complex link to poverty [23,24]. The availability and accessibility of health services may affect the population’s ability to settle health problems and thus affect the population’s poverty level [25]. Recently, debate on global health has increasingly focused on the importance of the health system and strengthening it [26]. Various studies have attempted to explore how to strengthen health systems in terms of equity and efficiency, mainly using the Gini coefficient, Lorentz curve, concentration index, and Theil index to study the fairness of health system distribution [27,28,29]. Others use Data Envelopment Analysis (DEA) or DEA optimization methods to study the efficiency of the health-care system [30,31,32]. Most studies show the existence of significant barriers to patient access and utilization of health services and large interprovincial differences in health-care delivery [33,34].

Scholars have also conducted many studies on the utilization of health services. Compared with individuals with high income, low-income individuals not only have less health system coverage, but they are also more likely not to seek medical care when they are sick [35]. When illness occurs, patients are more likely to make higher-level choices of health-service institutions [36]. The situation is similar in the United States, where only 12% of hospitalizations, 11% of days of care, and 6% of hospitalizations are provided by rural hospitals, despite 17% of the population living in rural areas [37].

**Figure 1:**
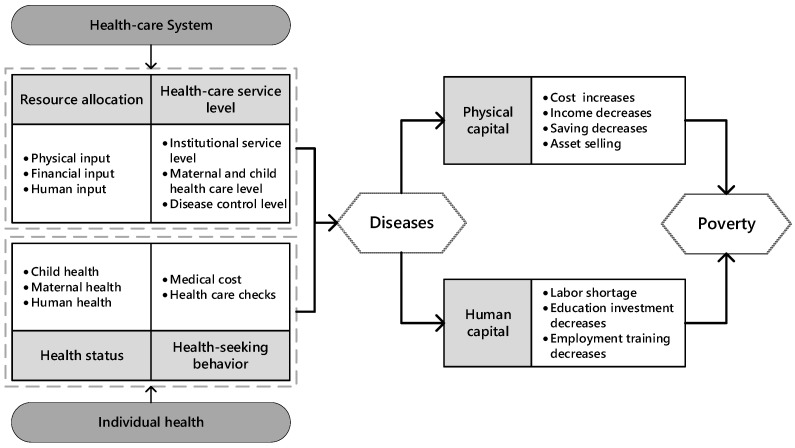
Theoretical framework of the impact of multidimensional health levels on poverty.

Although it is recognized that weak health systems exacerbate poverty, discussions on how health systems affect poverty remain limited. Thus, there is a strong need to integrate individual health and system health to analyze their role in poverty. By drawing on previous research, this study sketches a conceptual framework to present the impact on poverty from a multidimensional health perspective, as shown in Figure 1. In this framework, the impact on rural poverty is analyzed mainly from two aspects: health system factors and individual factors. This is performed through four indicators—health system inputs, health system service capacity, individual health status, and individual health-seeking behavior. When illness occurs, the short-term discounting of physical capital and the long-term increase in human costs may contribute to the occurrence or exacerbation of poverty. The empirical framework that follows explores these four indicators’ role in alleviating rural poverty.

## 2. Materials and Methods

### 2.1. Variable Selection and Description

The most common measure of poverty is an objective absolute poverty line set by governments, academics, or some organizations. For example, the World Bank set the global poverty line, which can be compared across the country [1]. In addition, many countries and regions tend to set a national poverty line (income poverty line or consumption poverty line) based on their economic and social realities and adjust it according to the level of national development, as is the case in China. The Chinese government defines the proportion of the population living below the poverty line as the incidence of poverty and takes this as the goal of poverty alleviation. From 2010 to 2020, China was committed to eradicating absolute poverty in rural areas, and the incidence of rural poverty has dropped from 2.8% to 0%. The incidence of poverty can only represent a small number of people in rural areas, and it is difficult to reflect the poverty situation there fully in recent years. How to measure rural poverty in China may be an important policy agenda for China’s rural areas after 2020 [38]. The proxy indicator of rural poverty in the current study is the Engel coefficient of rural residents. Engel proposed the Engel coefficient in 1857, defined as the ratio of food expenditures to total expenditures. Food is a necessity for a household, and as income increases, more money is spent on non-food items; thus, a lower share of food expenditure means a richer household, and a higher share of food means a poorer household [39]. The Engel curve lays the cornerstone of modern consumer behavior, economic analysis, and welfare analysis, and can reflect the poverty level of residents to a certain extent. The Engel coefficient has important policy implications. Orshansky firstly deduced the poverty line according to the Engel curve and influenced the poverty policy of the United States [40]. In sum, the Engel coefficient of rural residents not only reflects the living standards and changes in rural households comprehensively and accurately, but it is also more in line with the reality of China.

Combined with the theoretical analysis framework, four independent variables are selected to measure the health level of rural residents. Considering the completeness of the indicator system and the availability of data, the authors identified 24 secondary indicators to measure these four variables after searching provincial health statistics and excluding unavailable or incomplete indicators, as shown in Table 1. In China, the health system mainly consists of the state and administrative agencies at all levels providing health-care services to individuals. The service is shared by all the region’s residents; therefore, this study has adopted regional health service indicators instead of rural health system indicators.

Of the control variables, many indicators are related to changes in rural poverty. Generally speaking, the level of urbanization, government support, industrial structure, international communication, education level, and resource endowment are the main factors influencing rural poverty, which can aggravate or relieve rural poverty. To this end, the urbanization rate (Urb), the proportion of transfer income (Transfer), the proportion of non-agricultural industries (Noagr), the degree of openness (Open), per capita education age (Logyedu), and population density (Logpid) have been selected as control variable indicators which deflate the data.

The details of these variables are shown in Table 2.

### 2.2. Study Area and Data Resource

Considering the availability of data and unit for health policy development and implementation, the current study uses 31 provinces and autonomous regions of China as the spatial unit and 2012–2017 as the time unit for analysis. Three types of data are used. The first is provincial health-care data, including total health expenditures, number of health institutions, and per capita medical expenses. All data are extracted from the China Health Statistical Yearbook 2013–2018, provided by the National Health Commission of China. The second is provincial socioeconomic data, including gross domestic product, years of education per capita, urbanization rate, and total import and export trade. All socioeconomic data were obtained from the China Statistical Yearbook, provided by the National Bureau of Statistics of China. The third data type is GIS shapefiles, including points of interest in 2012–2017, and area boundaries from the National Basic Geographic Information System website. Table 3 shows the descriptive statistics after data processing.

### 2.3. Methods

#### 2.3.1. Processing Data

1. Calculation of distance of medical institutions

The coordinates model of the gravity centers and distance formulas are used to calculate distance. The specific formula used to calculate the health-care gravity centers is as follows:

(1) $$x=\frac{1}{n}\overset{n}{\underset{i=1}{\sum }}\cos\;x_{i}*\cos\;y_{i}$$

(2) $$y=\frac{1}{n}\overset{n}{\underset{i=1}{\sum }}\cos\;y_{i}*\sin\;x_{i}$$

(3) $$z=\frac{1}{n}\overset{n}{\underset{i=1}{\sum }}\sin x_{i}$$

(4) $$X=\frac{\arctan\left(\frac{y}{x}\right)*180{}^{\circ}}{\pi }$$

(5) $$Y=\frac{\arctan\left(\frac{z}{\sqrt{x^{2}*y^{2}}}\right)*180{}^{\circ}}{\pi }$$

In Formulas (4) and (5), *i* represents the *i*th health-care point and *n* represents the total number of health-care points. *X* and *Y* represent the health-care gravity centers in the calculation area and *x_i_* and *y_i_* represent the longitude and latitude of each health-care point in this area.

The moving distance of the center of health-care institutions’ gravity is an important measurement index for analyzing the center of gravity’s evolution trajectory. According to the Haversine formula:

(6) $$hav_{j}=hav\left(Y_{j}-Y_{j^{0}}\right)+\cos\left(Y_{j}\right)*\cos\left(Y_{j^{0}}\right)*hav\left(X_{j}-X_{j^{0}}\right)$$

(7) $$Hav=\overset{m}{\underset{j=1}{\sum }}hav_{j}*pop_{j}$$

In Formula (6), *j* represents the *j*th research unit, *X_j_* and *Y_j_* represent the health-care gravity centers of a county, and $X_{j^{0}}$ and $Y_{j^{0}}$ represent the longitude and latitude of the administrative center of a sub-region. Using Formula (7), weighted by the resident population of the county, the distance of the total area, *Hav*, is calculated. Taking a county as a unit to calculate the center of gravity helps explore the change of gravity center distance of each province more accurately.

2. Standardization of data

The dimensionless data can be represented as:

(8) $$x_{ij}\left(t_{k}\right)=\frac{x_{ij}^{*}\left(t_{k}\right)-min\left\{x_{ij}^{*}\left(t_{k}\right)\right\}}{max\left\{x_{ij}^{*}\left(t_{k}\right)\right\}-min\left\{x_{ij}^{*}\left(t_{k}\right)\right\}}\times 100$$

where $x_{ij}^{*}\left(t_{k}\right)$ represents the original observed value of the *j*th evaluation index of the *i*th evaluation object at the moment *t*(*k*), where *t*(*k*) = 2012, 2013, …, 2017; *i* = 1, 2, 3,…, *n*; and *j* = 1, 2, 3, …, *m*. $max\left\{x_{ij}^{*}\left(t_{k}\right)\right\}$, $min\left\{x_{ij}^{*}\left(t_{k}\right)\right\}$ are the maximum and minimum values of the *j*th indicator in n evaluated objects during 2012–2017. The value of a parameter after standardization is represented by $x_{ij}\left(t_{k}\right)$.

3. Measurements of multidimensional health level

The current study uses the vertical and horizontal layer-by-layer scatter degree method for multidimensional health level measurement. This method is a dynamic measurement of panel data that builds a time series three-dimensional data table, processing the underlying data layer by layer from the bottom up, calculating the weights, and obtaining the composite index [41]. The basic principle of this method is as follows:

There are n evaluated objects *O*_1_, *O*_2_, *O*_3_, …, *O_n_*, and each evaluation object has m evaluation indexes. Suppose the constructed evaluation index system has n large systems (target layer), which are recorded as *S_i_* (*i* = 1, 2, 3, …, *n*). Each large-scale system has *p* layers, and there are *n_p_* subsystems of the same level in each layer.

First, the subsystem index weight is determined. According to the index observation data set of the *q*th subsystem of layer P-1 (secondary layer) in the year *t_k_*, the comprehensive evaluation value of the *q*th subsystem of layer P-1 is:

(9) $$y_{i}^{\left(p-1,q\right)}\left(t_{k}\right)=\overset{n_{p}}{\underset{j=1}{\sum }}\omega_{j}x_{ij}^{\left(p-1,q\right)}\left(t_{k}\right)$$

To maximize the difference between the evaluation objects on the time series three-dimensional data table, it is therefore characterized by maximizing the sum of squares of deviations of $y_{i}^{\left(p-1,q\right)}\left(t_{k}\right)$. After standardizing the original data, we obtain $\bar{y}$= 0; hence,

(10) $$\sigma^{2}=\sum_{k=1}^{N}\sum_{i=1}^{n}{\left[y_{i}\left(t_{k}\right)-\bar{y}\right]}^{2}=\sum_{k=1}^{N}\sum_{i=1}^{n}{\left[y_{i}\left(t_{k}\right)\right]}^{2}=\sum_{k=1}^{N}\left[\omega^{T}H_{k}\omega \right]=\omega^{T}\sum_{k=1}^{N}\left[H_{k}\omega \right]=\omega^{T}H\omega$$

where *ω* = (*ω*_1_, *ω*_2_, *ω*_3_, …, *ω_m_*)*^T^*, *k* = 1, 2, 3, …, *N*, $H=\sum_{k=1}^{N}H_{k}$, and $H_{k}=A_{k}^{T}A_{k}$,

(11) $$A_{k}=\left(\begin{array}{ccc} x_{11}\left(t_{k}\right) & \cdots & x_{1m}\left(t_{k}\right)\\ \vdots & \ddots & \vdots \\ x_{n1}\left(t_{k}\right) & \cdots & x_{nm}\left(t_{k}\right) \end{array}\right),\;k=1,2,3,\ldots ,N$$

If *ω^T^ ω* = 1 is limited, the weight solving problem is transformed into solving the following nonlinear programming problems:

(12) $$\max\omega^{T}H\omega \;s.t.\{\begin{array}{c} -x,\;x<0\\ x,\;x\geq 0 \end{array}$$

When *w* is the (standard) eigenvector corresponding to the maximum eigenvalue of *H*, *σ*^2^ is the maximum, and max_‖*w*‖_ *ω^T^Hω* = *λ*_max_(*H*).

Second, the index weight of the parent system is determined. According to Formula (9), the evaluation value $y_{i}^{\left(p-1,q\right)}\left(t_{k}\right)$ is calculated, which is equivalent to the value of the parent system corresponding to layer P-2 (primary layer). Thus, the comprehensive evaluation value of the *r*th subsystem of the layer P-2 system is:

(13) $$y_{i}^{\left(p-2,r\right)}\left(t_{k}\right)=\overset{n_{p}}{\underset{q=1}{\sum }}\omega_{q}y_{j}^{\left(p-1,q\right)}\left(t_{k}\right)$$

The method determining *ω_q_* is consistent with the above.

Finally, the index weight of each parent system is iterated forward and then determined. The above steps are repeated up to the target layer, so the comprehensive evaluation value of the first layer system is:

(14) $$y_{i}^{\left(1,1\right)}\left(t_{k}\right)=\overset{n_{p}}{\underset{q=1}{\sum }}\omega_{q}y_{j}^{\left(2,q\right)}\left(t_{k}\right)$$

The results of indicator weights are shown in Table 1.

#### 2.3.2. Construction of Statistical Models

According to the first law of geography [42], everything is correlated to other things, but near things are more correlated than far things. Consequently, if the spatial analysis is neglected when performing econometric modeling, biased results will be generated [43], and spatial models should be considered to solve this problem. The Spatial Durbin Model (SDM) is a benchmark model that takes the spatial effects of the dependent variable into account, as well as the spatial autocorrelation of the independent variables. In this model, the single-stage lag term of the explanatory variable is incorporated into the model. It is defined in the following equation:

(15) $$Y=\rho WY+\beta X+\theta WX+\varepsilon ,\varepsilon \sim N(0,\delta^{2})$$

where *Y* is the dependent variable, *X* is the independent variable, ρ is the spatial autoregressive coefficient that measures the spatial correlation of the dependent variables, *β* is the coefficient of the independent variable, *θ* is the spatial regression coefficient that measures the spatial correlation of the independent variables, *W* is the spatial weight matrix, *WX* is the vector of the spatial lag independent variable, *WY* is the vector of the spatial lag dependent variable, and *ε* is the random disturbance term, which is usually considered to be independently distributed.

When the spatial dependence among the explanatory variables appears critical to the model and leads to spatial correlation, *θ* = 0, which is the spatial lag model (SLM); when the error terms of the model are spatially correlated, θ=−ρβ, which is the spatial error model (SEM).

Before building the spatial measurement model, it is necessary to determine the spatial weight matrix *W*. This study chose the widely used binary continuity matrix. The binary continuity matrix is defined by whether two units are neighbors. If unit *i* and unit *j* are contiguous, *W_ij_* = 1; if unit *i* and unit *j* are noncontiguous, *W_ij_* = 0.

## 3. Results

### 3.1. Spatial Econometric Model Testing

Moran’s I index was analyzed to evaluate the applicability of the spatial econometric model. This index detects the degree of spatial autocorrelation in rural poverty among China’s provinces. As shown in Table 4, the values of Moran’s I index were significant at 1% and greater than 0, indicating a positive spatial autocorrelation in rural poverty among 31 provinces. Hence, a spatial model should be used in this study.

Under certain conditions, SDM can be simplified to SLM or SEM, making it general or specific [44]. Based on the general SDM, the first step was to test whether the spatial panel data model was suitable and whether the SDM could be simplified to the SLM or SEM. The Lagrange multiplier (LM) test, the robust LM test, and the likelihood ratio (LR) test were used to check the model. Second, the Hausman test was adopted to examine the fixed-effects model against the random-effects model. The results of the LM test, the robust LM test, the LR test, and the Hausman test are presented in Table 5.

The results showed that when using the LM test, SLM and SEM passed the test at the 1% level. In the robust LM test, the null hypothesis of no spatial lag effect was rejected, and no spatial error effect was accepted, which implied that spatial dependence is highly important when undertaking econometric analysis. Simultaneously, the LR test results are significant at the 1% level, which indicates that the SLM and SEM should be abandoned, and the SDM model should be employed. The LR test result suggests the SDM model with spatial and time fixed-effects would degenerate into an SDM model with spatial fixed-effects (*p* = 0.1461). Further, the Hausman test indicated that a fixed-effects model was appropriate rather than a random-effects model. In general, the SDM model with spatial fixed-effects is most appropriate to explain the relationship between multidimensional health levels and rural poverty.

### 3.2. Spatial Econometric Regression Results

The empirical results of the SDM are presented in Table 6. The spatial parameter ρ is significantly positive at the 1% level, which implies the existence of spatial dependence among provinces. Specifically, changes in rural poverty in neighboring provinces would impact rural poverty in the central province. The main reasons for this phenomenon are the existence of frequent interregional trade and labor mobility, competitive relations, and the tendency to imitate development strategies between regions.

Le Sage and Pace suggest that when the coefficient of the spatial lagged term of the explained variable is significantly non-zero, the use of the SDM coefficients to measure the spillover effects of the explanatory variable will lead to systematically biases [45]. Therefore, this study decomposes the impact of multidimensional health levels on rural poverty changes in the SDM model into direct effects and indirect effects, as shown in Table 7. The direct effects represent the impact on poverty due to changes in the explanatory variable in local areas. The indirect effects indicate the impact on poverty due to changes in the explanatory variable in nearby areas. The total effects refer to the averaged impact on the poverty in all areas and consist of the sum of the direct and indirect effects. We will focus on analyzing four core independent variables.

1. The direct effect of health system input (X1) is significantly negative at the 10% level, suggesting that every 1% increase in health system input will directly result in a 0.0414% increase in the local area’s poverty. The direct effects differed from the estimated coefficient of −0.0295 due to the generation of feedback effects. As a result of these feedback effects, the impacts on other locations come back to the areas from which the impacts originated. Additionally, the indirect effect is significantly negative: a 1% increase in health system input in a given area will lead to a 0.171% decrease in poverty in nearby areas. The total effect is also significantly positive at the 1% level. These results indicate that health system input will positively affect rural poverty alleviation in this province and the neighboring provinces.
2. At the 1% level, the direct effect of the service capacity of medical resources (X2) is positive, which means that a 1% increase in health system services capacity (X2) in a given area will lead to a 0.105% increase in poverty. This shows that the increased capacity of health-care resources does not relieve rural poverty, but rather has a negative effect. The explanation of this interesting finding may be rooted in the inequality in health system services.
3. The direct effect of the individual health status (X3) was negative at the 10% level, suggesting the measurement of health in the province could decrease rural poverty by 0.0579%, but there was no spillover effect on neighboring provinces. This can be explained by the fact that the residents’ improved health status represents the effective improvement of the individual health level, which can lessen the frequency of illness and prolong the working hours of rural residents, thus effectively reducing expenditures on health services, increasing income, and contributing to the improvement of rural poverty.
4. At the 5% level, the direct effect of individual health-seeking behavior (X4) was negative, which manifests that every 1% increase in the index of health-seeking behavior of residents in this province will reduce rural poverty by 0.0151%. This can be interpreted as an increase in health-seeking behavior of residents that represents an enhancement in the attention they put on health, which may manifest itself in the form of health check-ups and the adoption of healthy lifestyle habits. This may allow them to effectively prevent disease and reduce the likelihood of illness, which in turn reduces the depreciation of physical and human capital and ultimately alleviates poverty. Similarly, individual health-seeking behavior had no spillover effect on surrounding provinces.

The control variables are also worth discussing in this context. The direct effect of non-agricultural industry share (Noagr) is 0.446 at a 1% level. This shows that the upgrading and optimization of industrial structure may occur mainly in cities, and their dividends for economic development and people’s lives are more concentrated in cities, which do not effectively contribute to rural development, and rural groups benefit less [46]. The direct effect of transfer income share of rural residents (Transfer) is significantly positive, which may be due to the inequality of income redistribution instruments in China. The direct, indirect, and total effects of per capita education age (Logyedu) are significantly negative. This result supports previous studies. The improvement of education and enhancement of knowledge and skill reserves enable rural populations to have better opportunities for employment and income growth, which have a positive effect on alleviating rural poverty [47]. The urbanization level, openness, and population density (Logpid) all have a significant positive effect on rural poverty reduction in neighboring provinces. This result may be due to the development of the region’s urban process and foreign communication, which attract migrant workers from surrounding areas to work, which then has a positive impact on their local rural income.

## 4. Discussion

### 4.1. Reflections on Theoretical Framework and Policy

This study put forward the concept of multidimensional health levels and included the social health system, which is closely related to residents’ poverty. The current framework is also helpful for other countries to explore the solution to health poverty. The analysis presents new evidence that multidimensional levels of health have a different impact on rural poverty. It also demonstrates that poor health and poverty work through multiple pathways, providing a guide on how to break the health–poverty cycle. Moreover, the level of health varies from province to province [48,49]. Therefore, while each has its own focus, improving individual health and system health should be performed simultaneously to alleviate rural poverty effectively.

Unlike other studies that measure health-care accessibility from an individual perspective [50], the current study joins a health-care system perspective and analyzes its inputs and utilization with consistent results. This new perspective similarly demonstrates the relationship between health systems and poverty, where health system inputs can reduce rural residents’ health expenditures and difficulties accessing health services [51]. Thus, health system inputs have a positive effect on rural poverty alleviation. Unlike other studies that used sample survey data [52], the current study used provincial statistics to measure individual health-seeking behavior and obtained consistent findings. Individual health-seeking behavior reflects the degree to which individuals value their health. When people pay more attention to healthy lifestyles, they may reduce the incidence of long-term chronic diseases and reduce the number of visits and treatment expenditures. The effects of these behaviors are more significant in lower social status groups [53], so individuals’ health-seeking behaviors positively affect poverty alleviation. However, further research is needed to explore how policymakers can increase the importance of health among rural residents [54]. Improvements in individual health status have a mitigating effect on rural poverty, which is consistent with a large body of existing research [55,56]. The current study also found that it has the largest effect on poverty alleviation in this framework. This can be explained by the fact that increases in the importance of individual health and health system investment positively affect health improvement [25,57], and individual health status has a cumulative amplification effect.

Health system service capacity is detrimental to alleviating rural poverty, which may be related to its unequal utilization. Health delivery systems face the challenge of maintaining revenues and providing affordable basic health care to the population. Both central and local policies have been adopted to improve the efficiency and equity of health services, but they are often distorted by additional factors, which are yet to be evaluated. Indeed, there is a serious mismatch between supply and demand for health services in China, with frequent overcrowding of large hospitals and unattended primary care facilities. This disparity has negatively impacted health inequities and rural poverty. The COVID-19 outbreak also exposed the wide variation in the quality of primary health-care services in China, insufficient total health resources, irrational outcomes, and inefficient primary services [58]. These are the main reasons why Chinese rural residents are less satisfied with primary health-care services [58,59,60]. Currently, the effect of the hierarchical diagnosis and treatment system in directing rural patients to primary health services is not significant, and there is a large gap with China’s goal of directing patients to primary health services [36,61]. Moreover, the limited role played by social medical insurance may be another explanation. China proposed the New Rural Cooperative Medical Scheme (NRCMS) in 2003 to make health care more affordable for the rural population, but scholars are divided on the program’s effectiveness [49]. Although there is government and social financial support, the NRCMS only covers a small part of rural patients’ medical expenses [62,63,64], and they still have to pay a large proportion of out-of-pocket expenses. Further, while the NRCMS provides a larger reimbursement rate for inpatient services, many studies have found a greater burden of outpatient expenditures for residents, especially for chronic diseases [65].

### 4.2. Research Contributions and Limitations

To the authors’ knowledge, this study is the first to integrate the effects of individual and systemic health, focusing on the relationship between health systems and rural poverty, helping to identify the complex relationship between health and poverty, and reflecting on how to strengthen global health systems. Another contribution is this study’s consideration of spatial spillover effects. The analysis demonstrates that health system inputs in another province also positively affect rural poverty alleviation in a neighboring province, which provides new directions for improving the efficiency of poverty reduction.

However, certain limitations of the study are also acknowledged. Firstly, although the Engel coefficient can reflect the level of and changes in the living of farming households, the coefficient does not fully reflect the level of rural poverty and it would be better if there were more scientific indicators to measure poverty in rural China. Secondly, the number of indicators for measuring individual health levels is relatively limited, which may lead to insufficient accuracy in measuring health levels. Future studies need to utilize more in-depth health data in the analysis, such as the prevalence of chronic diseases. Thirdly, this study analyzes the relationship between different dimensions of health levels and rural poverty, but will all secondary indicators impact rural poverty? This study did not discuss these questions, but they deserve to be pursued in future studies.

## 5. Conclusions

The eradication of poverty is a common mission of humanity. The issue of “health poverty” has plagued humankind for almost a thousand years, and even today, with advanced medical technology, it is still the subject of tireless exploration by scholars. Existing research has been conducted on different individual factors, demonstrating the role of disease in influencing poverty. However, what role does the health system play in this process? Reflections on this topic will help governments and societies develop better health-care reform policies to improve rural poverty. This study integrates a health–poverty conceptual framework and uses the case of China. Four dimensions of health level were defined and measured, including health system inputs, health system service capacity, individual health status, and individual health-seeking behavior. This was to specifically analyze the mechanism of the effect of health levels and rural poverty. Within this framework, this study used health system and rural poverty data from 31 provinces to quantify the impact of health on changes in rural poverty by applying a spatial model. The results suggest that increasing health system inputs, improving individual health status, and strengthening individual health-seeking behavior contribute to the alleviation of rural poverty across provinces. However, it also finds that an increase in health system service capacity exacerbates rural poverty, which may result from spatial disparities in the supply and demand of health services. This study has stressed the important but less-recognized role of the health system. Although several policies to improve the pro-poor effect of the health system have been employed in China, their performance appears to be ineffective. Therefore, it is important to consider both individual and systemic factors of health levels when designing strategies for rural development. Moreover, long-term efforts should be taken to alleviate the rural poverty effect by promoting supply-side reform of the health system.

## Figures and Tables

**Table 1:** Indicators for measuring multidimensional health levels.

Target Level Indicators	Primary Indicators	Secondary Indicators	Indicator Properties	Weight
Health system input (X1)	Manpower input	No. of health technicians per 1000 population	+	0.1
		No. of professionals per 1000 population	+	0.09
		No. of registered nurses per 1000 population	+	0.13
	Physical input	Accessibility distance of medical institutions (km)	-	0.24
		Fixed assets per bed (10 thousand yuan)	+	0.09
		No. of beds in medical and health institutions per 1000 population	+	0.2
		Number of tertiary hospitals per million population (piece/million people)	+	0.1
	Financial input	Total health expenditure per capita (yuan)	+	0.02
		Percentage of medical aid expenditure (‰)	+	0.04
Health system service capacity (X2)	Physician service capacity	Daily inpatient bed days by physicians (days)	+	0.05
		No. of consultations per day by physicians	+	0.05
	Hospital service capacity	Average days of hospitalization (days)	-	0.11
		Hospital bed utilization rate (%)	-	0.11
		No. of visits to medical institutions per capita	+	0.11
	Maternal and child health-care level	Rate of postnatal visits (%)	+	0.09
		Neonatal visitation rate (%)	+	0.08
	Disease prevention and control level	Incidence rate of class A and B ^1^ legally reported infectious diseases (1/100,000)	+	0.19
		Mortality rate of Class A and B legally reported infectious diseases (1/100,000)	+	0.22
Individual health status (X3)	Health level of women	Maternal mortality rate (1/100,000)	-	0.42
	Health level of infants and children	Proportion of children under 5 years old with moderate to severe malnutrition (%)	-	0.18
		Infant mortality rate (‰)	-	0.2
	Health level of residents	Mortality rate (‰)	-	0.2
Individual health-seeking behavior (X4)	Level of attention	No. of people having health check-ups in medical and health institutions	+	0.2
	Willingness to pay	Percentage of rural residents’ health-care expenditure (%)	+	0.8

^1^ Based on Law of the People’s Republic of China on the Prevention and Treatment of Infectious Diseases, Class A and B represent 27 infectious diseases such as plague, cholera, viral hepatitis, and dysentery.

**Table 2:** Variables for analysis.

Variable	Unit	Symbol	Measurement	Interpretation
Urbanization rate	%	Urb	Urb = Urban resident population/Total resident population	An indicator that measures the structure of urban and rural.
Proportion of non-agricultural industries	%	Noagr	Noagr = 1 − (Value added by the primary sector/GDP)	The changes in the proportional relationships among industries.
Proportion of transfer income	%	Transfer	Transfer = Transfer income of rural residents/Disposable income of rural residents	An indicator that measures the impact of policies on residents’ income.
Degree of openness	%	Open	Open = Total exports and imports of goods/GDP	Advanced foreign technology and foreign trade boost economic development.
Average years of school attainment per person	Year	Logyedu		An indicator that measures educational level.
Population density	Persons/sq. m	Logpid	Logpid = Resident population/Total area	Population density affects the cost of access to resources and employment for residents.

**Table 3:** Descriptive statistical analysis of variables.

Variable	Obs.	Mean	Std. Dev.	Min	Max
Pov	186	0.348	0.062	0.253	0.536
Urb	186	0.562	0.131	0.237	0.896
Noagr	186	0.901	0.050	0.76	0.996
Transfer	186	0.155	0.065	0.053	0.292
Open	186	0.249	0.271	0.014	1.254
Logyedu	186	2.425	0.101	2.07	2.683
Logpid	186	5.316	1.491	0.941	8.256

**Table 4:** Spatial correlation test of rural poverty.

Year	Moran’s I	Z Value	*p* Value
2012	0.465	4.613	0.000
2013	0.321	3.346	0.000
2014	0.325	3.503	0.000
2015	0.312	3.363	0.000
2016	0.266	2.955	0.002
2017	0.295	3.171	0.001

**Table 5:** Diagnostic test results.

Determinants	Statistics	Determinants	Statistics
LM test spatial lag	18.037 ***	LR test spatial lag	55.39 ***
Robust LM test spatial lag	1.021	LR test spatial error	80.64 ***
LM test spatial error	32.726 ***	Hausman test	113.83 ***
Robust LM test spatial error	15.710 ***		

Notes: *** *p* < 0.01.

**Table 6:** Results of SDM estimation with spatial fixed effects.

Variable ^1^	Main	Wx	Variable	Main	Wx
X1	−0.0295	−0.0911 ** ^2^	Noagr	0.399 ***	0.156
	(0.0219)	(0.0366)		(0.147)	(0.318)
X2	0.100 ***	0.00743	Transfer	0.108 ***	−0.236 ***
	(0.0345)	(0.0644)		(0.0415)	(0.0568)
X3	−0.0570 *	−0.00749	Open	−0.0270	−0.129 ***
	(0.0310)	(0.0565)		(0.0303)	(0.0433)
X4	−0.0152 **	0.00606	Logyedu	−0.212 ***	−0.372 ***
	(0.00592)	(0.0123)		(0.0667)	(0.126)
Urb	0.0234	0.521 *	Logpid	0.259 **	−1.086 ***
	(0.136)	(0.289)		(0.139)	(0.281)
ρ	0.419 ***				
	(0.0787)				
Log-likelihood	558.1				
Observations	186				

^1^ X1, X2, X3 and X4 represent respectively health system input, health system services capacity, individual health status, and individual health-seeking behavior. ^2^ *** *p* < 0.01, ** *p* < 0.05, * *p* < 0.10.

**Table 7:** Direct, indirect, and total effects for the spatial fixed SDM.

Variable	Direct Effect	Indirect Effect	Total Effect
X1	−0.0414 *	−0.171 ***	−0.212 ***
	(0.0218)	(0.0570)	(0.0610)
X2	0.105 ***	0.0849	0.190
	(0.0346)	(0.112)	(0.125)
X3	−0.0579 *	−0.0548	−0.113
	(0.0298)	(0.0872)	(0.0953)
X4	−0.0151 **	−3.99 × 10^−5^	−0.0151
	(0.00622)	(0.0205)	(0.0234)
Urb	0.0847	0.847 *	0.931 *
	(0.138)	(0.470)	(0.527)
Noagr	0.446 ***	0.552	0.998
	(0.165)	(0.567)	(0.675)
Transfer	0.0857 **	−0.298 ***	−0.212 ***
	(0.0400)	(0.0687)	(0.0674)
Open	−0.0449	−0.231 ***	−0.276 ***
	(0.0314)	(0.0798)	(0.0952)
Logyedu	−0.259 ***	−0.742 ***	−1.000 ***
	(0.0681)	(0.194)	(0.232)
Logpid	0.144	−1.578 ***	−1.433 ***
	(0.135)	(0.476)	(0.499)

Notes: *** *p* < 0.01, ** *p* < 0.05, * *p* < 0.10.

## Data Availability

The data that support the findings of this study are openly available via the National Bureau of Statistics of PRC at https://data.stats.gov.cn/easyquery.htm?cn=C01 (accessed on 16 March 2022), reference number [66].
